# Axillary metastases following total laryngectomy: an under-recognised pathway

**DOI:** 10.1017/S0022215126104836

**Published:** 2026-08

**Authors:** Eyal Yosefof, Ofir Zavdy, Tara Coreanu, Noga Kurman, Hanna Bernstine, Roee Noy, Yotam Shkedy, Amit Ritter

**Affiliations:** 1 Department of Otorhinolaryngology and Head and Neck Surgery, Rabin Medical Center-Beilinson Hospital, Petah Tikva, Israel; 2 Gray Faculty of Medical and Health Sciences, Tel Aviv University, Tel Aviv, Israel; 3 Davidoff Cancer Center, Rabin Medical Center, Petah Tikva, Israel; 4 Department of Nuclear Medicine, Rabin Medical Center, Petah Tikva, Israel; 5 Department of Otorhinolaryngology and Head and Neck Surgery, Rambam Health Care Campus, Haifa, Israel; 6 Rappaport Faculty of Medicine, Technion-Israel Institute of Technology, Haifa, Israel

**Keywords:** Laryngeal Neoplasms, Laryngectomy, Lymphatic Metastasis, Axilla, Squamous Cell Carcinoma of Head and Neck

## Abstract

**Objectives:**

Axillary lymph node metastases represent a rare and under-recognised pattern of disease progression in laryngeal squamous cell carcinoma, with limited data regarding their timing and clinical significance.

**Methods:**

We retrospectively reviewed all patients treated for advanced-stage laryngeal squamous cell carcinoma at two tertiary medical centres between 2017–2022, identifying cases that subsequently developed axillary lymph node metastases.

**Results:**

Five patients (3.6 per cent) developed axillary lymph node metastases, all following total laryngectomy, four after salvage surgery for recurrent disease. Mean interval from surgery to axillary lymph node metastases diagnosis was 9.9 ± 4.2 months. All tumours were moderately to poorly differentiated, and axillary involvement was frequently associated with mediastinal nodal disease. All patients received systemic therapy, including chemotherapy and/or immunotherapy, and three died of disease during follow-up.

**Conclusion:**

Axillary lymph node metastases occurred within a relatively narrow time window after total laryngectomy, suggesting a non-random metastatic pattern. Awareness of this pathway is important for post-laryngectomy surveillance and interpretation of follow-up imaging.

## Introduction

Axillary lymph node metastases (ALNM) represent a rare presentation of distant spread in head and neck squamous cell carcinoma (SCC), occurring in 2–9 per cent of cases^1^^,^^2^. Few case reports and series document ALNM from head and neck cancer, most commonly originating from thyroid malignancies^3^^–^^6^. Other rare origins include laryngeal^7^^,^^8^, oral cavity^9^^–^^11^, oropharyngeal^12^^,^^13^ and nasal cavity cancers^14^. Due to the rarity of ALNM, no standard treatment guidelines exist, and prognosis remains uncertain.

The axillary region is not routinely included in elective neck dissection or radiotherapy fields for laryngeal SCC (though contiguous with neck level 4). Consequently, clinically occult ALNM may be underestimated^15^. Delayed metastatic presentation in laryngeal cancer, particularly to the lungs, is well documented, often associated with advanced primary disease in the supraglottic region^16^^,^^17^. However, data specifically on ALNM in laryngeal cancer patients are scarce^7^^,^^8^, and treatment decisions (surgery, radiotherapy, or systemic therapy) are typically case specific due to limited evidence.

This study aims to describe a series of patients from our institutions who presented with ALNM following total laryngectomy for laryngeal SCC. We will detail primary tumour characteristics, timing and presentation of metastases, treatment approaches and outcomes.

Only isolated case reports and one small series have documented axillary spread of laryngeal SCC so far, leaving its incidence, timing and clinical correlates poorly defined. Consequently, ALNM remain an under-recognised manifestation of disease progression, with unclear implications for post-treatment follow-up and management. In this multicentre retrospective cohort, we describe the largest series to date of patients who developed ALNM following total laryngectomy for laryngeal SCC. We aimed not only to describe this rare metastatic pathway, but to highlight its implications for post-laryngectomy surveillance and clinical decision making.

## Materials and methods

### Data collection

This multicentre retrospective cohort included all patients with histologically confirmed laryngeal SCC treated by total laryngectomy for either primary or recurrent disease, or advanced-stage (stage III–IV) laryngeal SCC treated definitively by non-surgical means, including radiotherapy with or without chemotherapy, at two university-affiliated tertiary care centres between January 2017 and February 2022. Data were collected from patients’ medical records and included demographics, risk factors, clinical and pathological features, disease staging, treatment modalities and outcomes. Specific data related to recurrence after primary treatment were gathered, including timing of diagnosis, recurrence location (local, regional or distant), imaging findings, recurrence management and prognosis.

### Treatment and follow-up

All patients underwent pre-treatment cross-sectional imaging (either positron emission tomography-computed tomography (PET-CT) or computed tomography (CT) of the head, neck, and chest) to exclude distant metastasis and involvement of axillary or mediastinal lymph nodes. Management pathways for both primary and recurrent disease, including definitive (chemo)radiotherapy or total laryngectomy with or without adjuvant treatment, were finalised during multi-disciplinary team discussions. Follow-up baseline imaging, typically PET-CT, was conducted 10–12 weeks post-treatment. In cases with negative post-treatment imaging, patients were followed clinically in alignment with the National Comprehensive Cancer Network (NCCN) guidelines^18^, involving physical examinations at recommended intervals: every one to three months in the first year, every two to six months in the second year, every four to eight months in years three to five, and annually thereafter. Additional imaging was performed at the discretion of the treating surgeon or oncologists if symptoms or physical examination findings indicated potential recurrence. In cases where imaging suggested recurrence, biopsy was considered to confirm or rule out the diagnosis. For the rare occurrence of suspicious axillary lymph nodes, biopsy decisions were made on a case-by-case basis, usually by the managing oncologist.

Tumour staging followed the eighth edition of the American Joint Committee on Cancer (AJCC) staging system for laryngeal cancer^19^. Clinical follow-up involved a comprehensive medical history and head and neck examination by our multi-disciplinary team, following timelines recommended by contemporary NCCN guidelines.

## Results and analysis

### Clinical characteristics of the study population

The study cohort included 140 patients, comprising 51 surgical patients and 89 non-surgical patients, with a median age of 65 years (range, 42–94 years). Demographic and clinical characteristics are summarised in Table 1. The primary tumour site was the laryngeal glottis in 58 patients (41.4 per cent) and the supraglottis in 74 patients (52.9 per cent). Subglottic tumours were rare, observed in one patient (0.7 per cent). Transglottic tumours were identified in four patients (2.9 per cent), while hypopharyngeal tumours were observed in three patients (2.1 per cent). Out of the surgically treated subgroup, 13 patients (25.5 per cent) underwent total laryngectomy as the primary treatment for advanced-stage disease, while 38 patients (74.5 per cent) underwent salvage laryngectomy for persistent or recurrent disease. Neck metastases were present in 11 patients (21.5 per cent). Neck dissection, either unilateral or bilateral, was performed in 46 patients (90 per cent), and thyroid surgery (either lobectomy or total thyroidectomy) was conducted in 49 patients (96 per cent). Reconstruction was required in 41 patients (80.5 per cent), utilising a pectoralis major flap in 40 patients and an anterolateral thigh (ALT) free flap in one patient.

**Table 1.** Clinical characteristics of the study population Table 1 long description.

Characteristic	Surgical patients (no. of patients = 51)	Non-surgical patients (no. of patients = 89)
Median age, year (range)	63 (48−82)	66 (42−94)
Risk factors		
Alcoholism, *n* (%)	9 (17.5)	13(14.5)
Smoking, *n* (%)	46 (90)	79 (89)
Pack-years, mean ± SD	68.5 ± 32.5	68 ± 41
Primary tumour subsite, *n* (%)		
Glottis	32 (62.5)	26 (29)
Supraglottis	15 (29.5)	59 (66.5)
Subglottis	0	1 (1)
Transglottic tumour	3 (6)	1 (1)
Hypopharynx	1 (2)	2 (2.5)
Procedure, *n* (%)		
Primary total laryngectomy	13 (25.5)	
Salvage laryngectomy	38 (75.5)	
Unilateral neck dissection	34 (66.5)	
Bilateral neck dissection	11 (21.5)	
Thyroidectomy (lobectomy or total)	49 (96)	
Pectoralis major flap reconstruction	40 (78.5)	
Free flap reconstruction	1 (2)	
Specimen and histopathology, *n* (%)		
Subglottic extensions	17 (33.5)	
Neck metastases	11 (21.5)	
Tumour differentiation*		
Well	5 (10)	
Moderate	20 (39)	
Poor	24 (47)	
Adverse features		
Involved margin	6 (12)	
Extranodal extension	9 (17.5)	
Perineural invasion	23 (45)	
Lymphovascular invasion	10 (19.5)	

* Data was not available for two patients.

SD = standard deviation.

### Axillary lymph node metastases

**Figure 1.**
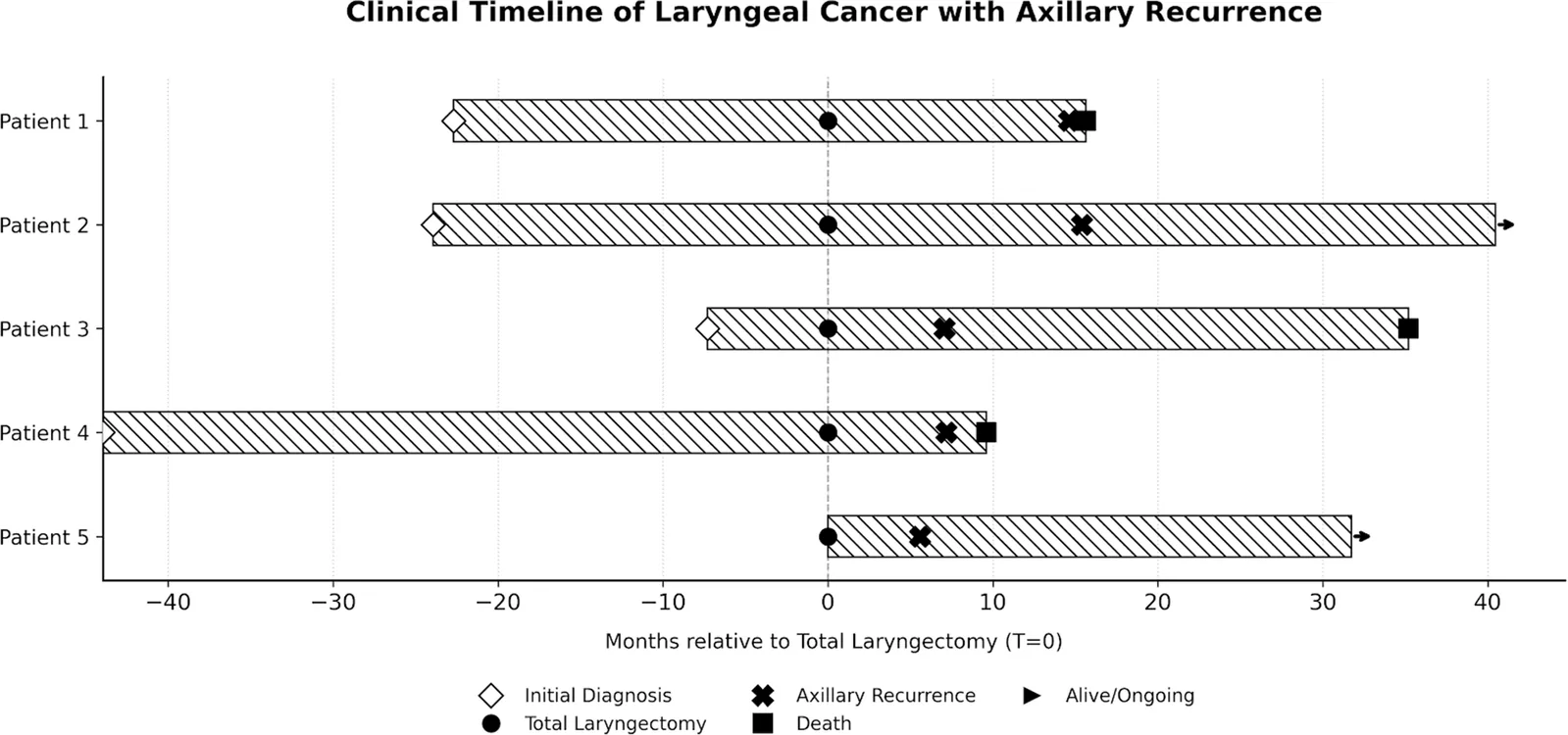
Swimmer’s plot illustrating the temporal course of ALNM; ALNM = axillary lymph node metastases following total laryngectomy.

Positron emission tomography imaging detected ALNM in five patients (3.6 per cent) during post-treatment follow-up, three of which were confirmed by biopsy (Table 2). All patients were in the surgically treated subgroup, and no patients treated with definitive radiation or chemoradiation experienced ALNM. The average interval from surgery to ALNM diagnosis was 9.9 ± 4.2 months. The temporal relationship between initial diagnosis, total laryngectomy, axillary relapse and clinical outcome for each patient is illustrated in Figure 1. All five patients had moderate to poorly differentiated glottic cancer. Four of these patients underwent salvage laryngectomy for recurrent laryngeal carcinoma, with pathology confirming negative resection margins and no evidence of neck metastases.

**Table 2.** Patients with ALNM after total laryngectomy Table 1 long description.

	Age	Smoking	Alcohol	Primary/Rec.	Tumour site	Primary tumour stage	Neck dissection	Recon.	Tumour staging at TL	Subglottic extension	Grade	Adverse features	Mediastinal metastases	Follow-up time (months)	Post-treatment status
1	61Y	120PY	No	Rec.	Glottis	T3N0M0	Bil. 2−4	Pec. major	T4aN0M0	Yes	Mod–poor		Yes	9	Died
2	64.5Y	40 PY	Yes	Rec.	Glottis	T3N0M0	Lt. 2−4	Pec. major	T4aN0M0	Yes	Mod–poor	Close margin, LVI	No	35	Died
3	68Y	20 PY	No	Primary	Glottis	T4aN2cM0	Bil. 2−4, 6	No	T4aN2cM0	Yes	Mod–poor	ENE, LVI	Yes	50	Persistent disease
4	74Y	80 PY	No	Rec.	Glottis	T1N0M0	Rt. 2−4	Pec. major	T2N0M0	No	Mod–poor	PNI	Yes	15	Died
5	69Y	40 PY	No	Rec.	Glottis	T2N0M0	Rt. 2−4, 6	Pec. major	T2N0M0	No	Mod–poor		Yes	41	Persistent disease

ALNM = axillary lymph node metastases; Bil. = bilateral; ENE = extranodal extension; Lt. =left; LVI = lymphovascular invasion; Mod = moderate; NA = not available; NED = no evidence of disease; Pec. = pectoralis; PNI = perineural invasion; PY = pack-years; Rec. = recurrent; Recon. = reconstruction; Rt. = right; TL = total laryngectomy; Y = years.

**Figure 2.**
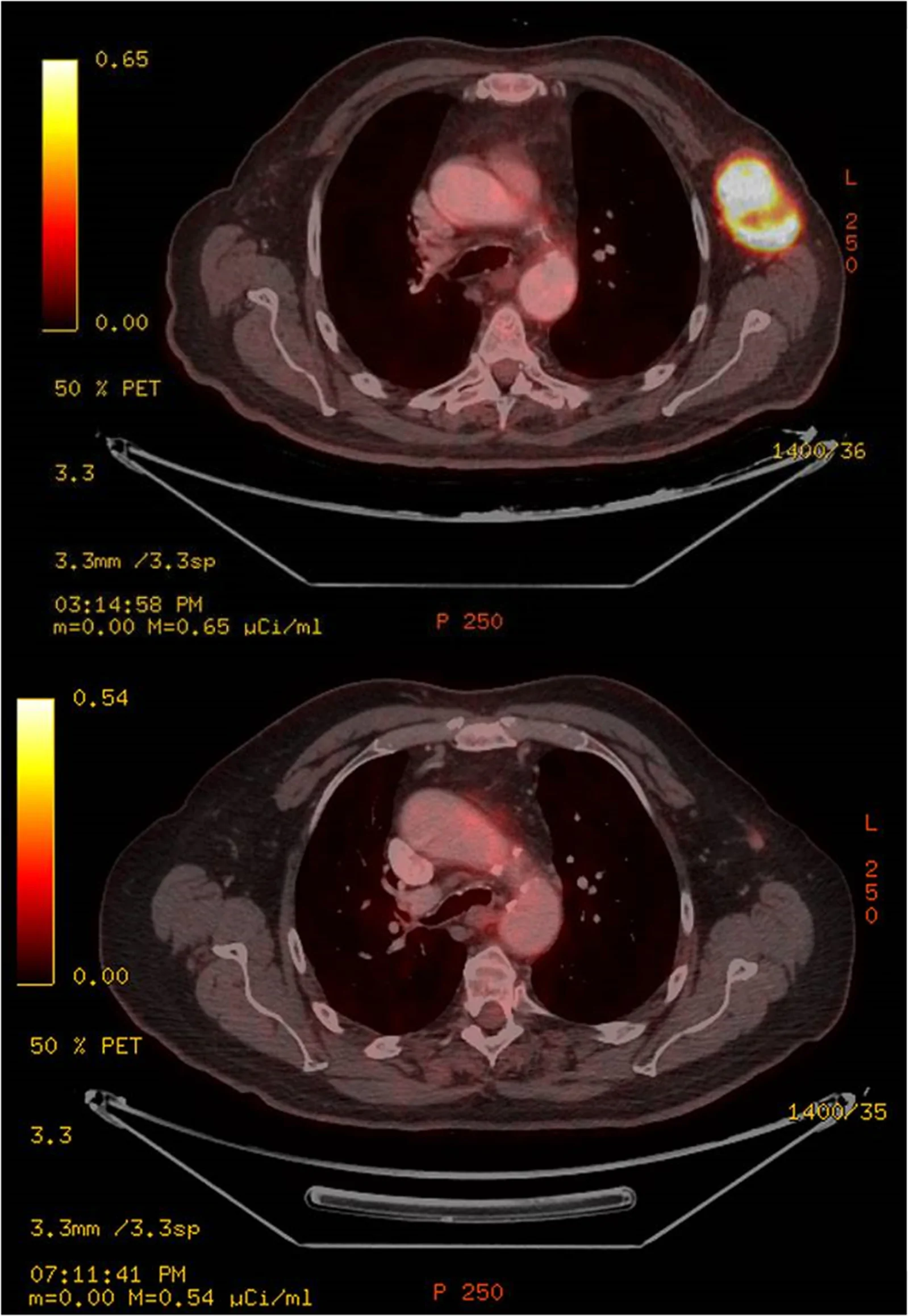
Sequential PET-CT scans of patient number 3. The upper panel demonstrates early axillary metastases detected two months after completion of adjuvant chemoradiotherapy following primary total laryngectomy. The lower panel, obtained two years later, illustrates complete and durable metabolic remission in the axilla after systemic therapy with pembrolizumab, cisplatin and 5-fluorouracil. The patient remains free of axillary disease fifty months after the primary surgery. Figure 2 long description.

For primary tumour treatment, 2 patients with early-stage glottic cancer (T1/2N0) received laryngeal radiation: 63 Gy in 28 fractions for T1N0 and 65.25 Gy in 29 fractions for T2N0. The other 2 patients with T3N0 glottic tumours received chemoradiotherapy, including 70 Gy in 35 fractions to the larynx, 50–54 Gy to the bilateral neck, and weekly cisplatin (40 mg/m^2^) for 7 weeks. The fifth patient developed ALNM following initial treatment, which included total laryngectomy and bilateral neck dissection for advanced laryngeal cancer. Histopathological examination revealed close surgical margins (1 mm) and bilateral level 2 neck metastases, with extranodal extension (ENE) on one side. The patient was treated with adjuvant chemoradiotherapy, receiving 66 Gy to the metastatic node with ENE, 60 Gy to the primary laryngeal site and bilateral neck, along with weekly cisplatin at a dose of 40 mg/m^2^ for a total of 7 weeks. Figure 2 illustrates the PET-CT scan demonstrating ALNM detected two months after completion of adjuvant chemoradiotherapy. Bilateral axillary adenopathy was observed in two patients, and the average size of the dominant lymph node was 2.6 cm. The mean maximum standardised uptake value (SUVmax) was 16.

Univariate analysis demonstrated a significant association between ALNM and the presence of mediastinal lymph node metastasis (*p* < 0.001) (Table 3). However, ALNM was not significantly associated with other factors, such as primary tumour subsite (notably, all ALNM cases originated from glottic tumours), subglottic extension, neck metastasis, flap reconstruction or adverse histopathological features. In multivariate analysis, no individual factor, including mediastinal lymph node metastasis, was independently associated with ALNM.

**Table 3.** Univariate analysis of factors associated with ALNM

Factor	No. of patients	No. of events (%)	Unadjusted odds ratio (95% CI)	*p*-value
Subglottic extension				
No	34	2 (6 %)	Reference	0.318
Yes	17	3 (17.5%)	3.43 (0.52, 22.84)	
Neck metastases				
No	40	4 (10%)	Reference	1.000
Yes	11	1 (9%)	0.9 (0.09, 8.98)	
Extranodal extension				
No	42	4 (9.5%)	Reference	1.000
Yes	9	1 (11%)	1.19 (0.11, 12.09)	
Mediastinal metastases				
No	45	1 (2%)	Reference	<0.001
Yes	6	4 (66.5%)	88 (6.47, 1196.20)	
Perineural invasion				
No	27	4 (15%)	Reference	0.354
Yes	24	1 (4%)	0.25 (0.03, 2.41)	
Lymphovascular invasion				
No	40	3 (7.5%)	Reference	0.292
Yes	11	2 (18%)	2.74 (0.4, 18.92)	
Resection margins				
Negative	39	4 (10%)	Reference	1.000
Positive	12	1 (8%)	0.79 (0.08, 7.88)	

ALNM = axillary lymph node metastases.

### Disease outcomes

Within the surgically treated subgroup, after a median follow-up of 25 months (range, 4 to 69 months), 16 patients (31.5 per cent) had died from their malignancy. Among the 5 patients who developed ALNM, 3 succumbed to the disease at a median interval of 15.5 months following total laryngectomy. Of these, two patients received chemoradiation and immunotherapy for metastatic spread, while one received only best supportive care. The remaining two patients with ALNM received systemic therapy for persistent disease, using pembrolizumab combined with either carboplatin and Taxotere® or cisplatin and 5-fluorouracil. Their follow-up periods were 50 and 41 months from the time of total laryngectomy to the last follow-up. Analysis revealed a significant association between ALNM and poor prognosis, as indicated by either mortality or persistent disease (*p* = 0.005).

### Statistical analysis

Differences in continuous variables between groups were analysed using the Mann–Whitney U test, while categorical variables were compared using Fisher’s exact test. Overall survival (OS) was defined as the time from the date of recurrence diagnosis to the date of death or last follow-up and was estimated using Kaplan-Meier survival analysis, with differences assessed by the log-rank test. All analyses were conducted using SPSS (version 26.0, IBM, Armonk, New York, USA).

## Discussion

This study describes a cohort of 140 patients with advanced or recurrent laryngeal SCC treated with either total laryngectomy or definitive radiation with or without chemotherapy, 5 of the total laryngectomy subgroup subsequently developed ALNM. To our knowledge, this represents the largest reported cohort of laryngeal cancer patients with ALNM to date.

Axillary lymph node metastases are most commonly associated with breast malignancy^20^. The occurrence of lymph node metastasis from head and neck primary tumours to the axilla is rare, with only small case series and reports documenting spread from thyroid^3^^–^^6^, oral cavity^9^^–^^11^, laryngeal^7^^,^^8^, oropharyngeal^12^^,^^13^ and nasal cavity^14^ tumours. Autopsy studies of patients who died from head and neck cancer report an axillary metastasis rate of 2–9 per cent^1^^,^^2^, though this may underestimate true incidence as routine axillary dissection is not typically performed in these patients^15^. Koch *et al*.^7^ reported a series of four patients with head and neck cancer and axillary metastasis, including three with primary laryngeal SCC and one with an unknown primary tumour. All patients were treated with surgery and radiotherapy for the primary tumour. Notably, each patient developed either a recurrence or a second primary head and neck malignancy before the onset of axillary nodal spread. This observation suggests that lymphatic manipulation during neck dissection or radiotherapy may change drainage pathways, possibly leading to axillary spread of recurrent or second primary head and neck cancers.

The jugular-subclavian angle represents a potential communication point between the cervical and axillary lymphatic systems, with normal lymphatic flow being centripetal toward this angle from both regions^3^^,^^10^^,^^21^. This normal flow can be altered by factors such as surgery, radiotherapy or the presence of lymphatic metastases, leading to retrograde flow from the angle toward the axillary lymph nodes, thereby explaining metastasis to the axilla. The lymphatic drainage from the axilla to the cervical nodes is also well documented in breast cancer patients. While axillary nodes are typically the first site of lymphatic drainage from the breast, supraclavicular metastases are observed in 1–4 per cent of cases, further highlighting the communication between the axillary and cervical lymphatic pathways^22^. In our cohort, only one patient had regional spread at the time of surgery. However, all patients underwent neck dissection along with total laryngectomy, including level 4 dissection, and three received radiotherapy to the neck, encompassing levels 2–4. Surgery or radiotherapy treatment to the lower neck may induce fibrosis at the jugular-subclavian angle, potentially redirecting lymphatic drainage towards the axillary pathway. Additionally, reconstruction with a pectoralis major flap, performed in four of five patients, involves manipulation of the lower neck area and may also contribute to the diversion of lymphatic drainage from the cervical to axillary nodes.

The mean time from surgery to the onset of axillary metastasis in our cohort was approximately 10 months. Oo *et al*.^10^ reported 3 cases of head and neck SCC with axillary metastasis, with a mean time to axillary spread of 21.3 months. Suehiro *et al*.^3^ described seven cases of thyroid carcinoma, with a mean time to axillary metastasis of 9.9 years. The discrepancy between the mean time in our cohort and that in Suehiro’s study is likely due to the more indolent nature of thyroid cancers compared to head and neck mucosal malignancies, particularly laryngeal SCC. The shorter time to axillary metastasis in our cohort, compared with Oo’s study, may be attributed to the aggressive histological features of our cases, as all patients had moderate to poorly differentiated tumours on pathology. The aggressiveness of tumours may also explain the significant association between mediastinal and axillary spread of disease (*p* < 0.001). Four patients had recurrent disease, three exhibited subglottic extension, and the patient who underwent primary total laryngectomy presented with regional spread (N2c stage), ENE and lymphovascular invasion on pathology.

Axillary lymph node metastases were significantly associated with poor prognosis in our cohort (*p* = 0.005), with three out of five patients succumbing to their disease and the remaining two others having persistent, likely non-curable disease. We recommend routine examination of the axillary area in advanced head and neck cancer patients, particularly those with recurrent or aggressive laryngeal carcinoma, especially if they have previously undergone neck dissection or radiotherapy to the cervical lymph node region. None of the patients in our study received surgical treatment for ALNM. Four patients had distant spread at the time of axillary metastasis diagnosis, and the fifth had severe treatment-related complications, including osteomyelitis of the skull base, which made surgery unfeasible. Interestingly, Nelson *et al*.^8^ reported a patient who, after undergoing total laryngectomy for recurrent laryngeal SCC, developed bilateral ALNM and was treated with bilateral ALNM. The patient survived for 24 years post-surgery. When ALNM is the sole manifestation of distant disease spread, surgery or other local therapy should be strongly considered to potentially improve patient prognosis.

Positron emission tomography imaging plays an important role in the detection of ALNM in post-laryngectomy patients. Given the mean diagnostic interval of less than ten months post-surgery, PET should be considered as part of routine surveillance, particularly in high-risk patients with aggressive tumour biology or recurrent disease. While PET is considered sensitive in identifying ALNM, biopsy confirmation remains the gold standard for definitive diagnosis. However, in cases where biopsy is not feasible, a high SUVmax combined with correlative imaging findings may provide sufficient diagnostic confidence. Given these findings, we recommend heightened attention to the axillary region during interpretation of routine PET-CT scans in patients with recurrent or aggressive laryngeal SCC, particularly following prior neck dissection or radiotherapy.

The limitations of this study include its retrospective design and small cohort size, consisting of only five cases of ALNM. However, as axillary metastasis is a rare occurrence in head and neck SCC, this represents, to our knowledge, the largest series of laryngeal SCC with ALNM. We believe that additional case series are needed to improve knowledge and data on this rare condition, thereby providing treating surgeons and oncologists with a better understanding of treatment options and prognosis.

## Conclusions

Axillary lymph nodes are an uncommon site of metastasis in laryngeal SCC, typically occurring in patients with recurrent disease following treatments like total laryngectomy with neck dissection. Recurrent laryngeal SCC is often aggressive, with concurrent mediastinal metastases being common. Patients with aggressive or recurrent laryngeal SCC who have undergone total laryngectomy should be routinely screened for ALNM. If ALNM is present, mediastinal involvement should be suspected, and imaging should be used to confirm or exclude it. Aggressive oncological treatment should be strongly considered, with surgery reserved for patients without other distant metastatic spread. Further studies and pooled analyses are needed to determine whether elective axillary imaging or local therapy can improve outcomes.

## Table 1 Long description

Clinical characteristics are compared between 51 surgical patients and 89 non-surgical patients, including age, risk factors, tumour subsite, and surgical pathology details. Median age was similar: 63 years in surgical patients and 66 years in non-surgical patients. Smoking was very common in both groups (90 percent surgical, 89 percent non-surgical), with similar average pack-years (about 68 in both), and alcoholism rates were also close (17.5 percent versus 14.5 percent). Primary tumour subsite differed: glottic tumours were more frequent in surgical patients (62.5 percent) than non-surgical patients (29 percent), while supraglottic tumours were more frequent in non-surgical patients (66.5 percent) than surgical patients (29.5 percent). Less common subsites included subglottic, transglottic, and hypopharyngeal tumours, each at low percentages in both groups. Procedure and histopathology details are reported only for the surgical group, including primary versus salvage laryngectomy, neck dissections, thyroidectomy, reconstruction type, subglottic extension, neck metastases, tumour differentiation, and adverse features such as involved margins and perineural invasion. Some values were unavailable for two patients, so percentages may not reflect the full sample for every characteristic.

Navigate back to Table 1.

## Table 2 Long description

Five patient cases with axillary lymph node metastases after total laryngectomy are summarized by demographics, exposures, tumour details, surgery, pathology risk factors, mediastinal spread, follow-up, and outcome. Ages ranged from 61 to 74 years, with heavy smoking histories from 20 to 120 pack-years; alcohol use was reported in one case. Four cases were recurrent glottic tumours and one was a primary glottic tumour. Subglottic extension was present in three cases, and tumour grade was moderate to poor in all patients. Neck dissection varied from unilateral levels 2 to 4 to bilateral levels 2 to 4 with or without level 6, and pectoralis major reconstruction was used in four cases. Adverse features were inconsistently reported, including close margin with lymphovascular invasion, extranodal extension with lymphovascular invasion, and perineural invasion, while two cases had no adverse features listed. Mediastinal metastases were noted in four of five patients. Outcomes were poor: three patients died and two had persistent disease, with follow-up ranging from 9 to 50 months; interpretation is limited by the small case series and some missing or blank entries.

Navigate back to Table 2.

## Figure 2 Long description

The upper panel demonstrates early axillary metastases detected two months after completion of adjuvant chemoradiotherapy following primary total laryngectomy. The lower panel, obtained two years later, illustrates complete and durable metabolic remission in the axilla after systemic therapy with pembrolizumab, cisplatin, and 5-fluorouracil. The patient remains free of axillary disease 50 months after the primary surgery.

Navigate back to Figure 2.

## References

[1] Gowen GF, Desuto-Nagy G. The incidence and sites of distant metastases in head and neck carcinoma. *Surg Gynecol Obstet* 1963;116:603–7 13949636

[2] Hoye RC, Herrold KM, Smith RR, Thomas LB. A clinicopathological study of epidermoid carcinoma of the head and neck. *Cancer* 1962;15:741–9 10.1002/1097-0142(196207/08)15:4<741::aid-cncr2820150408>3.0.co;2-8 14449431

[3] Suehiro A, Nagahara K, Moritani S, Omori K. Axillary lymph node metastases from thyroid carcinoma: report of seven cases. *Auris Nasus Larynx* 2021;48:718–22 10.1016/j.anl.2020.10.016 33172762

[4] Singh SS, Krishnan S, Tigapuram NK, Sood A, Satapathy S, Rohilla M et al. Axillary lymph nodal metastases from thyroid carcinoma: report of two cases with review of literature. *J Nucl Med Technol* 2021;49:89–91 10.2967/jnmt.120.249128 33020235

[5] Krishnamurthy A, Vaidhyanathan A. Axillary lymph node metastasis in papillary thyroid carcinoma: report of a case and review of the literature. *J Cancer Res Ther* 2011;7:220–2 10.4103/0973-1482.82936 21768721

[6] Nakayama H, Wada N, Masudo Y, Rino Y. Axillary lymph node metastasis from papillary thyroid carcinoma: report of a case. *Surg Today* 2007;37:311–5 10.1007/s00595-006-3395-2 17387564

[7] Koch WM. Axillary nodal metastases in head and neck cancer. *Head Neck* 1999;21:269–72 10.1002/(sici)1097-0347(199905)21:3<269::aid-hed13>3.0.co;2-q 10208671

[8] Nelson WR, Sisk M. Axillary metastases from carcinoma of the larynx: a 25-year survival. *Head Neck* 1994;16:83–7 10.1002/hed.2880160117 8125794

[9] Rayatt SS, Dancey AL, Fagan J, Srivastava S. Axillary metastases from recurrent oral carcinoma. *Br J Oral Maxillofac Surg* 2004;42:264–6 10.1016/j.bjoms.2003.12.004 15121278

[10] Oo AL, Yamaguchi S, Iwaki H, Amagasa T. Axillary nodal metastasis from oral and maxillofacial cancers: a report of three cases. *J Oral Maxillofac Surg* 2004;62:1019–24 10.1016/j.joms.2003.08.042 15278869

[11] Pandey R, Biswas R, Halder A, Pandey D. Carcinoma buccal mucosa with left axillary lymph node metastasis: first reported case and review of the literature. *J Cancer Res Ther* 2019;15:693–5 10.4103/jcrt.JCRT_49_18 31169243

[12] McKenzie BJ, Loock JW. Axillary nodal metastasis at primary presentation of an oropharyngeal primary carcinoma: a case report and review of the literature. *J Med Case Rep* 2009;3:7230 10.4076/1752-1947-3-7230 PMC2737770 19830142

[13] Cheng CY, Su TF, Lin YH, Ho CL, Huang WS. Rare axillary metastasis from squamous cell carcinoma of the tonsil. *Clin Nucl Med* 2013;38:e304–5 10.1097/RLU.0b013e318270833b 23478853

[14] Islam S, Cole CV, Hoffman GR, Brennan PA. Bilateral axillary metastasis from a primary ethmoidal squamous cell carcinoma. *J Laryngol Otol* 2006;120:353–5 10.1017/S0022215106000375 16553967

[15] Kowalski LP. Noncervical lymph node metastasis from head and neck cancer. *ORL J Otorhinolaryngol Relat Spec* 2001;63:252–5 10.1159/000055751 11408823

[16] Spector GJ. Distant metastases from laryngeal and hypopharyngeal cancer. *ORL J Otorhinolaryngol Relat Spec* 2001;63:224–8 10.1159/000055746 11408818

[17] Pan Y, Hong Y, Liang Z, Zhuang W. Survival analysis of distant metastasis of laryngeal carcinoma: analysis based on the SEER database. *Eur Arch Otorhinolaryngol* 2019;276:193–201 10.1007/s00405-018-5244-5 30542764

[18] National Comprehensive Cancer Network. *Head and neck cancers* (Version 1.2026). NCCN Clinical Practice Guidelines in Oncology 10.6004/jnccn.2020.0031 32634781

[19] Amin MB, Greene FL, Edge SB, Compton CC, Gershenwald JE, Brookland RK et al. The eighth edition AJCC cancer staging manual: continuing to build a bridge from a population-based to a more “personalized” approach to cancer staging. *CA Cancer J Clin* 2017;67:93–9 10.3322/caac.21388 28094848

[20] Carter CL, Allen C, Henson DE. Relation of tumor size, lymph node status, and survival in breast cancer cases. *Cancer* 1989;63:181–7 10.1002/1097-0142(19890101)63:1<181::aid-cncr2820630129>3.0.co;2-h 2910416

[21] Rouvière H. *Anatomie des lymphatiques de l’homme*. Paris: Masson; 1932

[22] Coopey SB. Supraclavicular and contralateral axillary lymph node involvement in breast cancer patients. *Ann Surg Oncol* 2022;29:6100–5 10.1245/s10434-022-12134-7 35794365

